# Altered Volume and Functional Connectivity of the Habenula in Schizophrenia

**DOI:** 10.3389/fnhum.2017.00636

**Published:** 2017-12-22

**Authors:** Lei Zhang, Hao Wang, Shuxin Luan, Shaojun Yang, Zhuo Wang, Jinhui Wang, Hua Zhao

**Affiliations:** ^1^Department of Physiology, College of Basic Medical Sciences, Jilin University, Changchun, China; ^2^Department of Radiology, The First Hospital of Jilin University, Changchun, China; ^3^Institutes of Psychological Sciences, Hangzhou Normal University, Hangzhou, China; ^4^Zhejiang Key Laboratory for Research in Assessment of Cognitive Impairments, Hangzhou Normal University, Hangzhou, China; ^5^Department of Clinical Psychology, The First Hospital of Jilin University, Changchun, China; ^6^Neuroscience Research Center, The First Hospital of Jilin University, Changchun, China

**Keywords:** habenular nucleus, schizophrenia, morphology, resting-state functional magnetic resonance imaging, functional connectivity

## Abstract

The pathogenesis of schizophrenia (SCH) is associated with the dysfunction of monoamine neurotransmitters, the synthesis and release of which are mainly regulated by a key structure, the habenular (Hb) nucleus. However, little is known regarding whether SCH is associated with structural or functional alterations in the Hb. In this study, we combined structural and resting-state functional magnetic resonance imaging to investigate the changes in volume and functional connectivity of the Hb in 15 patients with SCH vs. 16 age- and gender-matched healthy controls (HCs). Morphologically, the absolute volume of the bilateral Hb was significantly lower in the SCH patients than in the HCs. Functionally, the bilateral Hb showed significantly enhanced functional connectivity with the left medial prefrontal cortex (mPFC) in the SCH patients. Additionally, the SCH patients exhibited increased functional connectivity of the left Hb with the left lingual gyrus and right inferior frontal gyrus (IFG). A further exploratory analysis revealed that the SCH patients showed increased functional connectivity between the right Hb and several subcortical regions related to dopaminergic pathways, including the left ventral striatum, caudate and putamen. Finally, the increased functional connectivity of the right Hb with the mPFC was positively correlated with the Brief Psychiatric Rating Scale (BPRS) scores in the patients. Together, these results suggest that the altered volume and functional connectivity of the Hb may be involved in the pathogenesis of SCH and thus that the Hb may serve as a potential target in developing new therapeutic strategies in SCH.

## Introduction

Schizophrenia (SCH) is a complicated mental disorder characterized by severe dysfunctions of thought, affect and behavior (Andreasen, 1999). This disorder leads to persistent of hallucinations, delusions, apathy and cognitive disorders, as well as a lack of social skills, all of which seriously affect quality of life.

Previous studies have demonstrated that the dysfunction of monoamine neurotransmitters play an important role in the development of SCH. Among the various monoamine neurotransmitters, dopamine (DA) has recently attracted increasing attention due to its role in the pathogenesis of SCH (Guillin et al., 2007; Heinz and Schlagenhauf, 2010). It has been reported that positive symptoms, including hallucinations and delusions, are associated with DA hyperfunction in subcortical regions, such as the striatum, while negative symptoms, including apathy and loss of cognitive function, are related to DA hypofunction in the prefrontal lobe (Abi-Dargham, 2004). Given the crucial role of DA dysfunction in the pathogenesis of SCH, it is important to characterize brain regions that regulate the midbrain DA system in order to better understand SCH pathogenesis.

The habenula (Hb) is an epithalamic structure located in the dorsal diencephalic conduction system. It includes the medial habenula and lateral habenula (LHb) and provides an important link between the limbic forebrain and the midbrain regions (Sutherland, 1982; Andres et al., 1999). The Hb receives inputs via the stria medullaris from the basal ganglia and limbic system, including the entopeduncular nucleus, lateral hypothalamus, septum and medial frontal cortex (Araki et al., 1984; Hikosaka, 2010), and it projects to the midbrain structures, such as the interpeduncular nucleus, ventral tegmental area (VTA), substantianigra (SN), raphe nuclei and locus coeruleus via the fasciculus retroflexus (Scheibel, 1997; Hikosaka, 2010). Evidence from animal studies has shown that the Hb, particularly its lateral part, is a key area that controls dopaminergic neurons in the VTA and SN (Christoph et al., 1986). The excitation of the LHb exert an inhibitory effect on dopaminergic neurons in the SN and VTA indirectly by activating the GABAergic neurons either in the VTA/SN or in the rostromedial tegmental nucleus (Jhou et al., 2009). A study by Ji and Shepard (2007) showed that electrical stimulation of the LHb could inhibit the electrical activity of midbrain dopaminergic neurons. Moreover, the LHb is an important region that controls the activity of serotonergic neurons in the raphe nuclei and influences the function of the midbrain DA system by mediating the activity of raphe nucleus neurons. Recently, studies have also addressed the role of the Hb/LHb in higher brain functions such as reward, emotion, learning and memory, sleep and wakefulness (Yang et al., 2008; Shen et al., 2012; Zhao et al., 2015; Zhang et al., 2016; Li et al., 2017), dysfunctions of which are associated with the pathogenesis of SCH (Levesque et al., 2012; Lee et al., 2015; Ferrarelli and Tononi, 2017). For example, experiments in rats have shown that damage to the Hb not only leads to distraction (Lecourtier and Kelly, 2005) but also results in impaired learning and memory ability (Lecourtier et al., 2004). In addition, the Hb is reported to show a higher incidence of calcification in patients with SCH (Sandyk, 1992). All these findings suggest a critical role for the Hb in the pathogenesis of SCH. However, it remains largely unknown whether and how the structure and function of the Hb are changed in patients with SCH.

To address this gap, we combined structural and resting-state functional MRI to compare the volume and functional connectivity of the Hb between patients with SCH and healthy controls (HCs). Specifically, the functional connectivity analysis was performed in both a voxel-wise manner for an exploratory examination of whole-brain alterations and in a region-wise manner for a hypothesis-driven test for some specific regions of interest (ROIs). The regions included the bilateral SN/VTA, caudate, putamen and ventral striatum which were related to SCH (Guillin et al., 2007; Ellison-Wright et al., 2008; Yoon et al., 2013), and also be important downstream targets of the Hb. Given the important role of the Hb in regulating the DA system, the dysfunction of which is closely related to the pathogenesis of SCH, we characterized the SCH-related alterations of Hb in both brain structure and function to assess its potential as an imaging biomarker.

## Materials and Methods

### Participants

In total, 35 right-handed participants, including 18 patients with SCH and 17 age- and gender-matched HCs, were enrolled in this study. SCH was diagnosed by an experienced clinical psychiatrist and a trained interviewer according to the Diagnostic and Statistical Manual of Mental Disorders Fourth Edition (DSM-IV) criteria and excluded the presence of Axis I conditions in control participants (Mai et al., 2008). The patients (3–8 years since illness onset, mean illness duration of approximately 4.5 years) were recruited through the Department of Radiology, the First Hospital of Jilin University, Changchun, China. The HCs were recruited from the local area through poster advertisements. Exclusion criteria for all the participants included any serious medical or neurological conditions, drug/alcohol dependence history or abuse, serious head trauma, a positive urine drug screen on the day of the experiment, pregnancy or any MRI contraindications. Four participants were excluded from the final analysis because of image artifacts (one patient), excess head motion (two patients), or missing data (one HC). Therefore, 15 SCH patients and 16 HCs were ultimately included in this study. There were no significant differences in gender (SCH: nine males and six females, HCs: eight males and eight females; *P* = 0.576), age (SCH: 36.67 ± 10.18 years, HCs: 36.06 ± 12.38 years; *P* = 0.425) or educational level (SCH: 9.40 ± 3.54 years, HCs: 10.81 ± 2.99 years; *P* = 0.109) between the SCH patients and HCs. The detailed demographic and clinical data are summarized in Table 1. The study was approved by the ethics committee at the First Hospital of Jilin University and was conducted in accordance with the Declaration of Helsinki. Written informed consent was obtained from each participant.

**Table 1:** Demographic and clinical data for all participants.

	HCs (*N* = 16)	SCH (*N* = 15)	*P* values
Gender (M/F)	8/8	9/6	0.576
Age (years)	36.06 (12.38)	36.67 (10.18)	0.425
Education (years)	10.81 (2.99)	9.40 (3.54)	0.109
Disease duration (years)	-	6.13 (4.09)	-
PANSS total	-	92.00 (16.06)	-
Positive scale	-	23.53 (4.41)	-
Negative scale	-	21.00 (4.21)	-
General scale	-	47.47 (11.74)	-
BPRS total	-	39.40 (5.99)	-

*Data are represented as the mean (standard deviation). Gender data were analyzed with a χ^2^ test. Age and education data were analyzed with permutation tests. HCs, healthy controls; SCH, Schizophrenia; M, male; F, female; PANSS, Positive and Negative Syndrome Scale; BPRS, Brief Psychiatric Rating Scale*.

### Clinical and Neuropsychological Assessments

Demographic, clinical and neuropsychological characteristics, including age, gender, education, disease duration, the Positive and Negative Syndrome Scale (PANSS) and the Brief Psychiatric Rating Scale (BPRS), were recorded by an experienced neurologist (SXL, with more than 15 years of experience in clinical psychology). The healthy subjects were interviewed to confirm that there was no experience of psychiatric or neurological illness or a history of psychiatric diseases among their first-degree relatives.

### MRI Data Acquisition

All participants were scanned on a 3.0T GE Discovery MR750 (General Electric, Boston, MA, USA) using an eight-channel phased-array head coil with foam padding to minimize head motion. Structural and resting-state functional MRI images were acquired for all participants. During the whole scan, the participants were instructed to keep their eyes open and remain motionless as much as possible. The three-dimensional T1-weighted scans were acquired with a spoiled gradient-recalled acquisition in a steady-state (SPGR) sequence. The parameters were as follows: repetition time (TR) = 8.2 ms, echo time (TE) = 3.2 ms, slice thickness = 1.0 mm, voxel size = 1 × 1 × 1 mm^3^, field of view (FOV) = 256 × 256 mm^2^, matrix = 256 × 256, flip angle (FA) = 15°, NEX = 1 and 192 sagittal slices. The functional images were acquired with a T2*-weighted gradient-echo planar imaging sequence with the following parameters: TR = 2000 ms; TE = 30 ms, FA = 90°, voxel size = 3.75 × 3.75 × 3 mm^3^, FOV = 240 × 240 mm^2^, and 44 axial slices parallel to the AC-PC line. The functional images covered the whole cerebrum for all participants by visual inspection. However, the cerebellum was not covered fully for several participants. The functional scan lasted for 6 min and 180 volumes were obtained in total.

### Structural Imaging Analysis

#### Definition of the Hb Mask

In the current study, individual Hb masks were manually delineated based on their T1-weighted structural images with a human brain atlas for reference (Mai et al., 2008). This delineation was performed by an experienced neuroradiologist (ZL, with more than 10 years of experience in neuroradiology) who was blinded to the participants’ clinical states and using ITK-SNAP software^1^. To examine the reliability of the manual Hb masks, we randomly selected 20 participants (10 patients and 10 HCs) and manually delineated their Hb masks again. The dice coefficient and intraclass correlation coefficient were subsequently calculated to quantify intra-rater reliability in both shape and size, respectively, of the Hb masks.

#### Habenula Volume

For each participant, the absolute volume of the Hb was calculated as the number of voxels within the Hb mask multiplied by the size of a voxel (i.e., 1 × 1 × 1 = 1 mm^3^).

### Functional Image Analysis

#### Data Preprocessing

All resting-state functional MRI data preprocessing was implemented using GRETNA (Wang et al., 2015) based on the SPM12 software^2^. After discarding the first five volumes to allow for magnetic saturation, we conducted slice timing (sinc interpolation), head motion correction (six-parameter rigid-body transformation), spatial normalization into the Montreal Neurological Institute (MNI) space (via the transformation fields derived from tissue segmentation of individual structural images) and smoothing (Gaussian kernel with a 6-mm full width at half maximum). Two patients were excluded from further analysis according to the head motion criteria of >3 mm in displacement or 3° in rotation in any direction. Notably, given the small size of the Hb, the functional images were resampled to 2-mm isotropic voxels after the spatial normalization and the Hb ROIs were masked out before spatial smoothing to ensure sufficient functional specificity. The resulting images subsequently underwent removal of linear trends and band-pass filtering (0.01–0.1 Hz). Finally, the white matter, cerebrospinal fluid and head motion profile (24-parameter model; Friston et al., 1996) signals were regressed out from each voxel’s time series.

#### Seed-based Whole-brain Functional Connectivity

To conduct Hb-based functional connectivity analysis, individual Hb ROIs were first converted into the MNI space according to the deformation fields that were derived from the tissue segmentation of each participant’s T1-weighted image. All the resultant Hb ROIs were carefully inspected, and the mean MNI coordinates of all the participant-specific Hb ROIs were approximately [−4, −26, 2] for the left Hb and [6, −26, 2] for the right Hb. Then, the mean time series of the bilateral Hb was separately exacted and correlated with the time series of each voxel over the entire brain, which resulted in two whole-brain functional connectivity maps for each participant. Finally, a Fisher’s r-to-z transformation was applied to all the functional connectivity maps to improve the normality.

#### Exploratory Analysis of Functional Connectivity between the Hb and Subcortical Nuclei

Previous studies have demonstrated that changes in the structure and function of brain areas, such as the caudate, putamen, ventral striatum and SN/VTA, are associated with the pathogenesis of SCH (Camchong et al., 2011; Yoon et al., 2013), and the Hb shows close fiber connections and functional connectivity with these areas (Hennigan et al., 2015). However, in the current study, we did not observe any significant alterations in functional connectivity between these regions and the Hb (see “Results” section), possibly due to the small sample size and/or the relatively small sizes and low signal-to-noise ratios of these deep nuclei. We therefore performed an exploratory analysis to examine the functional connectivity between the bilateral Hb and the following eight subcortical ROIs (radius = 4 mm): the bilateral caudate ([−11/11, 5, 6]; Lawson et al., 2014), putamen ([−23/23, 18, −3]; Nielsen et al., 2016), ventral striatum ([−10/10, 8, −4]; Lawson et al., 2014) and SN/VTA ([−8/8, −20, −18]; Bunzeck and Düzel, 2006). Specifically, the Pearson correlation coefficients were calculated between the bilateral Hb and each of the eight subcortical ROIs with respect to their mean time series. Again, a Fisher’s r-to-z transformation was applied to all the resultant correlation coefficients to improve the normality.

### Statistical Analysis

A non-parametric permutation test (1000 permutations) was used to examine between-group differences in demographic variables (age and education), Hb volume and Hb functional connectivity with eight subcortical regions. Gender data were analyzed with a chi-square test. For whole-brain functional connectivity analysis, a voxel-wise one-sample *t*-test was first performed to determine brain regions that showed significant connectivity with the left and right Hb within each group, followed by between-group comparisons with a voxel-wise two-sample *t*-test. The 3dClustSim procedure was used to correct for whole-brain comparison of Hb functional connectivity, which was performed using the ANFI software^3^. Specifically, a mixed spatial autocorrelation function model was used in the 3dClustSim procedure, which efficiently controlled for false positive rates (Cox et al., 2017). The detailed parameters are as follows: primary height threshold of *P* < 0.005; extent threshold of *P* < 0.05 (corresponding to cluster size >280 voxels); estimated kernel smoothness = (10.666 mm, 11.502 mm, 10.964 mm); 1000 simulations; and corner connected. Finally, a non-parametric Spearman correlation was performed to test the relationship between the MRI-based Hb alterations and the patients’ clinical variables (disease duration, PANSS and BPRS). We did not perform multiple comparison correction for the correlation analysis given the small sample size and the exploratory nature of the current study.

## Results

### Reliability of the Hb Mask

**Figure 1:**
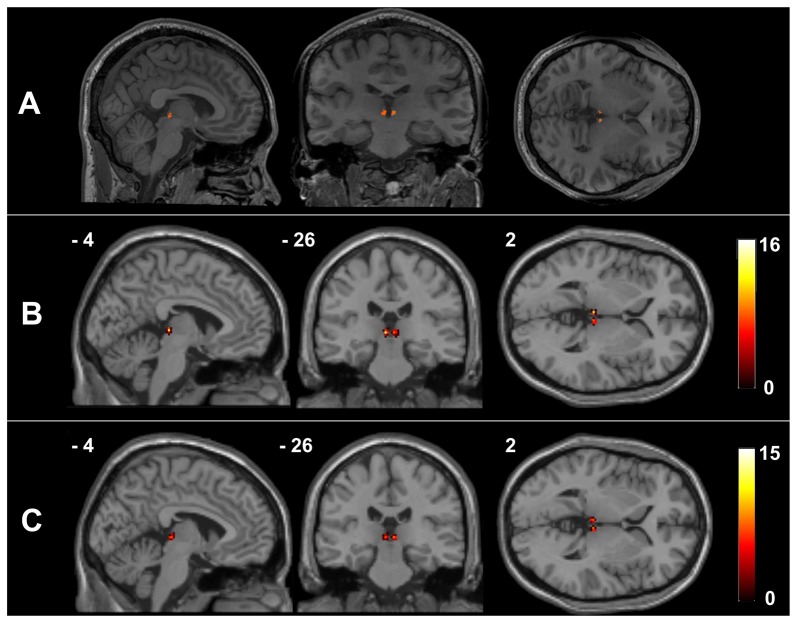
The habenula regions of interest (ROIs) in native and Montreal Neurological Institute (MNI) spaces. Individual habenula ROIs were first drawn manually on the high-resolution T1-weighted structural images in native space and are shown for a representative participant **(A)**. The resulting ROIs were then transformed from the native space into the MNI space by applying the deformation fields derived from the tissue segmentation of each participant’s T1-weighted image, after which they were summed over all the healthy controls (HCs) **(B)** and schizophrenia (SCH) patients **(C)** to demonstrate the consistency of the habenula locations.

Figure 1 presents the Hb mask of a representative healthy participant and a summary of the Hb probability maps of all participants in each group. The manual Hb masks showed high intra-rater reliability in both shape (dice coefficient = 0.939 ± 0.016 for the left Hb and 0.926 ± 0.048 for the right Hb) and size (intraclass correlation coefficient = 0.919 for the left Hb and 0.934 for the right Hb). These results indicate that the manual Hb masks were reliable for subsequent imaging analyses.

### Volume of the Hb

The absolute volumes of both the left Hb (HCs = 24.02 ± 3.20 mm^3^; SCH = 21.83 ± 2.16 mm^3^; *P* = 0.016) and right Hb (HCs = 20.42 ± 3.46 mm^3^; SCH = 18.27 ± 2.63 mm^3^; *P* = 0.025) were significantly smaller in the SCH patients than in the HCs. There was no significant difference in the total intracranial volume (TIV) between the two groups (*P* = 0.168). After controlling for individual TIVs, the left Hb showed a tendency toward lower volume in the SCH group than in the control group (*P* = 0.058), and the right Hb volume did not differ significantly between the two groups (*P* = 0.113).

### Resting-State Functional Connectivity of the Hb

**Figure 2:**
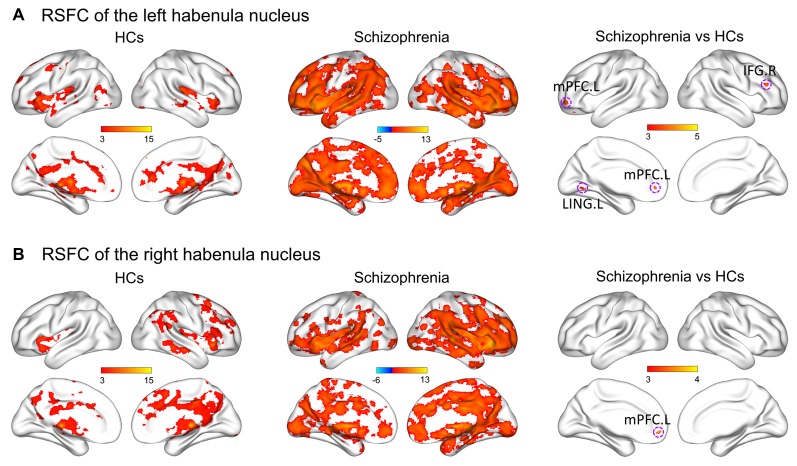
Within-group patterns and between-group differences of the RSFC for the bilateral habenula. RSFC of the left habenula nucleus **(A)** and RSFC of the right habenula nucleus **(B)**. The color bars represent the T scores. HCs, healthy controls; RSFC, resting-state functional connectivity; L, left; R, right; IFG, inferior frontal gyrus; LING, lingual gyrus; mPFC, medial prefrontal cortex.

Figure 2 shows within-group patterns and between-group differences in the resting-state functional connectivity (RSFC). For the HCs, the left Hb showed positive functional connectivity mainly with the thalamus, basal ganglia, ventral striatum, medial prefrontal cortex (mPFC), inferior frontal gyrus (IFG), superior temporal gyrus, temporal pole, cingulum, precuneus, lingual gyrus, hippocampus, amygdala, raphe nuclei, VTA and SN. Similar regions were found to show positive functional connectivity with the right Hb despite a more focal spatial distribution. Further, the bilateral Hb in the SCH patients was markedly larger and showed a markedly stronger functional connectivity than that in the HCs.

Further between-group comparisons revealed that compared with the HCs, the SCH patients had significantly greater functional connectivity of the left Hb with the left mPFC, left lingual gyrus and right IFG, as well as significantly greater functional connectivity of the right Hb with the left mPFC (*P* < 0.05, corrected; Table 2). No regions showed decreased functional connectivity in SCH patients.

**Table 2:** Regions showing different functional connectivity with the habenula (Hb) in patients with SCH.

Region	Voxels	Peak MNI coordinates			*t*-value	Cohen’s *d*
		*X*	*Y*	*Z*		
L Hb functional connectivity						
L Medial prefrontal cortex	878	−16	56	−2	4.080	1.118 (0.160)
R Inferior frontal gyrus	502	52	34	22	4.814	1.196 (0.153)
L Lingual gyrus	300	−28	−56	4	4.238	0.982 (0.098)
R Hb functional connectivity						
L Medial prefrontal cortex	324	−18	52	0	4.272	1.047 (0.158)

*Data are represented as the mean (standard deviation) for the Cohen’s d. L, left; R, right; MNI, Montreal Neurological Institute*.

### Functional Connectivity between the Hb and Subcortical Regions

**Figure 3:**
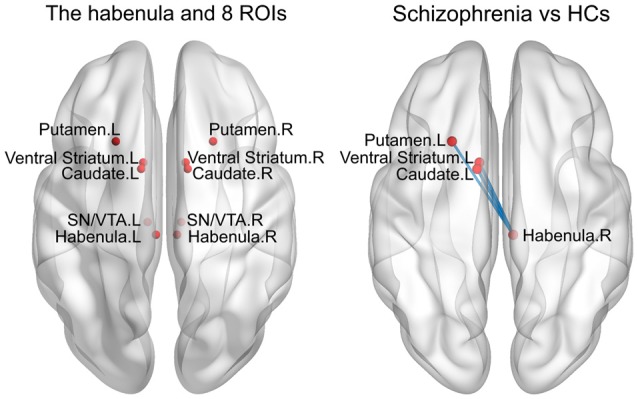
Enhanced RSFC between the habenula and subcortical regions in the SCH group. ROIs, regions of interest; HCs, healthy controls; L, left; R, right; SN/VTA, substantianigra/ventral tegmental area.

Compared with the HCs, the SCH patients showed significantly greater functional connectivity between the right Hb and the left caudate (*P* = 0.008), left putamen (*P* = 0.016) and left ventral striatum (*P* = 0.011; permutation test, False Discovery Rate corrected; Figure 3). There were no significant differences for other comparisons.

### MRI-Clinical Relationship

**Figure 4:**
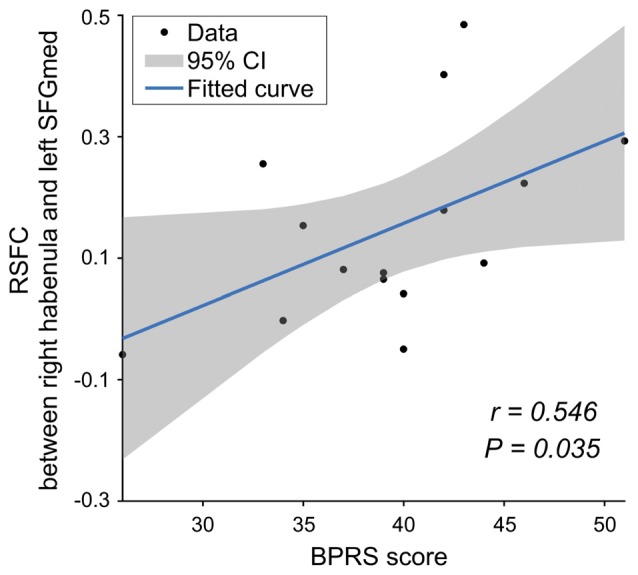
The relationship between the RSFC and clinical data for the SCH group. mPFC, medial prefrontal cortex; BPRS, Brief Psychiatric Rating Scale.

In the SCH group, there was a significantly positive correlation between the functional connectivity of the right Hb with the left mPFC and the BPRS scores (*r* = 0.546, *P* = 0.035; Figure 4). No significant correlations were found for other tests.

## Discussion

Although the volume of Hb is small, it has been the focus of an increasing number of investigations in psychiatric diseases. Dysfunction of Hb may lead to the development of SCH-like symptoms through mis-suppressed midbrain dopaminergic activity (Fakhoury, 2017). However, until now, little research has been performed to characterize the relationship between Hb and SCH. In this study, we utilized structural and resting-state functional MRI to investigate brain structural and functional alterations of the Hb in SCH. SCH-related decreases in absolute volume and increases in intrinsic functional connectivity were observed for both the left and right Hb. Moreover, the increased Hb functional connectivity correlated with the BPRS scores of the patients. These findings provide novel insights into the pathogenesis of SCH.

We found that the absolute volume of both the left and right Hb was smaller in the SCH patients, whereas the TIV was not significantly different between two groups. This finding is consistent with previous observations that compared with normal individuals, patients with SCH exhibited a higher proportion of epithalamic calcification (Sandyk, 1992) and lower capillary density in the bilateral Hb (Bernstein et al., 2016). The administration of antipsychotics for treating SCH has been shown to increase glucose metabolism in the LHb (Dedeurwaerdere et al., 2011), and these results further support our findings in the current study. The volume reduction, hyper-calcification and hypo-metabolism together suggest that the activity of the Hb may be reduced in SCH and that this dysfunction may lead to the development of SCH-like symptoms by enhancing midbrain dopaminergic activity (Sandyk et al., 1991). Additionally, the reduced volume of the Hb has also been reported in patients with behavioral variant frontotemporal lobe dementia (Bocchetta et al., 2016), which is associated with SCH-like hallucinations, delusions and cognitive and behavioral abnormalities. These results suggest that the Hb may play a role in SCH-related cognitive dysfunction, a model that is supported by experiments in rats showing that damage to the Hb results in a decreased ability to learn, memorize and be attentive, typical features of SCH (Lecourtier and Kelly, 2005). However, it is noteworthy that a postmortem study did not observe changes in the Hb volume of SCH patients at 12–48 h after death (Ranft et al., 2010). One possible factor that might contribute to this discrepancy is the marked differences in the age and disease duration of the SCH patients studied. Another possible reason for this difference may be that patients included in the postmortem study died of various causes that may confound the results and thus result in an inconsistent pattern.

It should be noted that no significant differences were found in the Hb volume between the SCH patients and HCs after controlling for individual TIVs. This indicates that the observed decrease in Hb volume in SCH patients may reflect a global effect. Nevertheless, a trend toward smaller relative Hb volume was noted in the SCH patients. Thus, it is possible that the non-significant difference in relative Hb volume may be due to the small sample size. Future studies are warranted to help clarify this point.

Interestingly, the above-mentioned trend was obvious only for the left Hb, which is consistent with a previous study showing that compared with the right Hb, the left Hb had more dramatic decreases in the quantity of ATP-binding cassette subfamily B member 1, which is involved in maintaining the steadiness of neural function in patients with SCH (Bernstein et al., 2016). The seemingly left-larger-than-right lateralized changes of the Hb volume may be due to the leftward lateralization of the globuspallidus in SCH (Okada et al., 2016), one of the most important upstream brain regions influencing Hb activity (Herkenham and Nauta, 1977).

Our functional connectivity analysis revealed that compared with the HCs, the patients with SCH exhibited greater coupling of the bilateral Hb with the left mPFC as well as of the left Hb with the right IFG. Recently, it has been proposed that dysfunction of the PFC leads to subcortical dopaminergic hyperactivity, and this theory has attracted tremendous interest in SCH research (Yoon et al., 2013). The findings in the current study support this theory by demonstrating a possible pathway through which the PFC mediates the effect of DA via strengthening its functional coupling with the Hb in SCH. Interestingly, we found a positive correlation between the Hb-mPFC functional connectivity and patients’ BPRS scores. This finding suggests the potential of using the Hb and related functional connectivity as biomarkers for assessing disease progression and as candidates for therapeutic targets in SCH. In addition to the mPFC and right IFG, the left Hb also showed increased functional connectivity with the lingual gyrus in the SCH patients. The lingual gyrus is a brain structure related to visual processing and logical conditions. It can be speculated that the increased functional connectivity between the Hb and the lingual gyrus may contribute to the hallucinatory symptoms observed in SCH patients. However, we did not test the hallucinatory symptoms of the patients, and further studies are needed to examine this speculation.

Our exploratory analysis of the Hb-subcortical functional connectivity revealed that only the right Hb exhibited increased coupling with the contralateral ventral striatum, caudate nucleus and putamen. The crossed pattern of abnormal functional connectivity has been observed previously for the association between the Hb and midbrain pathways during a reward feedback task (Hennigan et al., 2015). Together with the functional connectivity results discussed above, it seems that in a schizophrenic state, there are different priorities of functional connectivity alterations between the two sides of the Hb, with the right Hb mainly increasing its coupling with subcortical regions and the left Hb predominately increasing its coupling with cortical areas. The phenomenon of asymmetry in Hb functional connectivity was observed in a recent study showing enhanced functional coupling of the right Hb with the SN/VTA and increased functional coupling of the left Hb with the limbic system (Hétu et al., 2016). It is important for future studies to examine the asymmetry of the bilateral Hb with respect to the structural and functional alterations in these regions.

There are several limitations in the current study. First, all the SCH patients recruited in the current study were in a chronic disease state. Despite a wash-out period prior to this study, we cannot rule out possible effects of long-term use of antipsychotic medications on our findings. Future studies on treatment-naïve SCH patients are needed to clarify this issue. Second, recent studies suggested using stringent primary thresholds (e.g., *P* < 0.001) for cluster-extent based correction methods to ensure spatial specificity and to control false-positive rates (Woo and Moon, 2014; Eklund et al., 2016). However, we chose to use *P* < 0.005 as a primary threshold in the current study given the small sample size. Future studies are thus needed to test the reproducibility of our findings by employing a larger cohort of patients. Third, we were unable to distinguish the lateral and medial Hb in this study because of the small size of the Hb and the insufficient spatial resolution and contrast of our data. It would be interesting for future studies to examine the subtle and differential changes in different parts of the Hb in SCH by utilizing ultra-high field (e.g., 7.0 T) MRI scanners and advanced imaging sequences (e.g., multiband accelerated echo planar imaging). Finally, the current study examined only the changes in volume and functional connectivity of the Hb in SCH. Whether and to what extent SCH may affect other features of the Hb, such as microstructure, metabolism and cerebral blood flow, should be further investigated in future studies to develop a comprehensive characterization of SCH-induced Hb alterations by utilizing multimodal imaging techniques.

In conclusion, using structural and resting-state functional MRI, the current study demonstrated abnormal brain features of the bilateral Hb in patients with SCH as characterized by decreased absolute volume and increased functional connectivity, mainly with frontal and subcortical regions. Moreover, the altered Hb functional connectivity was related to the neuropsychological performance of the patients. These findings suggest possible contributions of the Hb to the pathogenesis of SCH, which will advance our understanding of SCH pathogenesis as well as the treatment of this disease.

## Author Contributions

HZ, JW and LZ: design of the experiments. LZ, SL and ZW: execution of the experiments. LZ, SL, SY and HW: collection and analysis of data. LZ, HW, JW and HZ: draft of manuscripts and preparation of figures. All the authors approved the final version of the manuscript.

## Conflict of Interest Statement

The authors declare that the research was conducted in the absence of any commercial or financial relationships that could be construed as a potential conflict of interest.

## Footnotes

^1^http://www.itksnap.org/pmwiki/pmwiki.php

^2^www.fil.ion.ucl.ac.uk/spm/software/spm12/

^3^http://afni.nimh.nih.gov/afni
